# Prostate-Associated Gene 4 (PAGE4): Leveraging the Conformational Dynamics of a Dancing Protein Cloud as a Therapeutic Target

**DOI:** 10.3390/jcm7060156

**Published:** 2018-06-17

**Authors:** Ravi Salgia, Mohit Kumar Jolly, Tanya Dorff, Clayton Lau, Keith Weninger, John Orban, Prakash Kulkarni

**Affiliations:** 1 Department of Medical Oncology and Therapeutics Research, City of Hope, Duarte, CA 91010, USA; tdorff@coh.org (T.D.); pkulkarni@coh.org (P.K.); 2 Center for Theoretical Biological Physics, Rice University, Houston, TX 77005, USA; mkjolly.15@gmail.com; 3 Division of Urology and Urologic Oncology, City of Hope, Duarte, CA 91010, USA; Cllau@coh.org; 4 Department of Physics, North Carolina State University, Raleigh, NC 27695, USA; krwening@ncsu.edu; 5 Institute for Bioscience and Biotechnology Research, University of Maryland, Rockville, MD 20850, USA; orban@ibbr.umd.edu; 6 Department of Chemistry and Biochemistry, University of Maryland, College Park, MD 20742, USA

**Keywords:** PAGE4, prostate cancer, symptomatic BPH, Cancer/Testis Antigen, ADT, IAD

## Abstract

Prostate cancer (PCa) is a leading cause of mortality and morbidity globally. While genomic alterations have been identified in PCa, in contrast to some other cancers, use of such information to personalize treatment is still in its infancy. Here, we discuss how PAGE4, a protein which appears to act both as an oncogenic factor as well as a metastasis suppressor, is a novel therapeutic target for PCa. Inhibiting PAGE4 may be a viable strategy for low-risk PCa where it is highly upregulated. Conversely, PAGE4 expression is downregulated in metastatic PCa and, therefore, reinstituting its sustained expression may be a promising option to subvert or attenuate androgen-resistant PCa. Thus, fine-tuning the levels of PAGE4 may represent a novel approach for personalized medicine in PCa.

## 1. Introduction

According to a relatively recent report, ‘An Aging World: 2015’ commissioned by the National Institute of Aging of the United States and produced by the US Census Bureau, the world’s elderly population continues to grow at an unprecedented rate. Almost 8.5% of the world’s population (617 million) is aged 65 and over, and this number is projected to mushroom to nearly 17% (1.6 billion) by 2050. Given that the incidence of both prostate cancer (PCa) and benign prostatic hyperplasia (BPH) increase with age, it is obvious that these diseases affect a large fraction of the elderly male population, and underscore the urgent need for effective therapies and strategies to treat and manage them. 

Here, we first briefly review the current treatment options for the two diseases and present evidence which indicates that PAGE4, a remarkably prostate-specific protein, can be a novel, multifaceted pharmacological target for these desperate maladies. Next, we examine the unique structural features of the PAGE4 protein ensemble (or more specifically, the lack of a specific structure). We discuss how the aberrant expression of PAGE4 and its conformational dynamics may enable this protein to contribute to the progression of BPH from an asymptomatic to a severe symptomatic form. Finally, we review data indicating PAGE4 may function both as an oncogenic factor in early-stage PCa and as a metastasis suppressor in advanced disease, and argue how its conformational malleability could be exploited as its Achilles heel.

### 1.1. Prostate Cancer 

PCa is a major global healthcare issue. In 2018, it is estimated that we will see ~165,000 new diagnoses and >29,000 deaths from the disease in the US alone [1]. Indeed, according to the American Cancer Society, it is the third leading cause of cancer-related death in men. Since the advent of the prostate-specific antigen (PSA) assay and improved detection of biopsy samples, metastatic disease at presentation has become a rarity in the urology clinic. However, in 2012 the United States Preventive Services Task Force recommended that healthy men should no longer be screened for PCa because the test does not save lives overall, and often leads to more tests and over-diagnosis. However, proponents continue to argue in favour of PSA-based screening and the debate continues [2,3,4,5]. 

Nonetheless, a continuing problem in urology today is the large number of patients with either abnormally high PSA/negative biopsies or patients with positive biopsies but low Gleason scores (≤6 with no Gleason 4) [6,7,8]. Many of the patients in this category who are over 60 years of age will undergo active treatment that is unlikely to extend their life span [9,10]. With no medical modalities to treat or prevent the disease in the early stages, this unsettled group is usually considered as low or very low risk for PCa by the clinicians. Patients in this group are, thus, advised to follow the active surveillance (AS) protocol [11,12]. This protocol involves close monitoring of PSA levels combined with periodic imaging and repeat biopsies with the intention of avoiding treatment unless there is evidence of disease progression [11,13,14].

Despite this cautious approach, however, the disease progresses in a significant fraction of these patients [15], and much to the chagrin of the urologists, they face an imminent danger of developing a high-risk disease. Indeed, it is estimated that as many as 40% [11] and up to 58% [16] of the patients under AS progress to “high-risk” (GS ≥7) PCa that can become metastatic and potentially turn lethal. In the recent *ProtecT* (Prostate Testing for Cancer and Treatment) trial [17], 1643 men were randomly assigned to radical prostatectomy, external beam radiation, or AS. The trial found that ~10% of patients in the AS group had developed metastatic disease or died of PCa at 10 years, thus underscoring the lurking danger for these patients.

Unfortunately, until recently, reliable biomarkers that can distinguish between high-risk PCa patients who are likely to progress to metastatic disease, from low-risk patients whose disease is unlikely to progress were not available to urologists. This lack of precision may have contributed to significant “overtreatment” of patients who would have otherwise required only conservative management [18]. However, the advent of advanced technologies and sophisticated bioinformatics algorithms has fueled the discovery of novel biomarkers that can be helpful for post-biopsy decision-making in low-risk patients and post-radical prostatectomy in selected risk groups [19]. Although intended to be used in combination with the accepted clinical criteria to stratify PCa according to biological aggressiveness and direct initial patient management, these new biomarkers are gaining acceptance. Despite the rising popularity, additional studies are still needed to investigate the clinical benefit of these new technologies, the financial ramifications and their utilization in the clinic. Notwithstanding these challenges, there are still no therapeutic options that can be offered to patients with low-risk PCa. 

On the other hand, the treatment course for patients with high-risk disease is definitive and is given with the intent to cure the cancer. Patients are typically recommended surgery followed by androgen deprivation therapy (ADT). However, many patients with non-metastatic yet androgen-resistant PCa (CRPC), an earlier stage of PCa, can experience disease progression despite ADT. Unfortunately, no Federal Drug Administration (FDA) approved treatment options are presently available for this condition until these patients develop metastatic disease. Notwithstanding the potential side effects of surgery [20] and the failure of ADT due to the emergence of CRPC in most patients pursuant to an initial positive response [21,22], the big question is how do we address androgen resistance in the future? Developing the so-called “next-gen” drugs to an old target (i.e., androgen receptor (AR)), even if they are more effective [23,24,25], are not likely to be a viable long-term solution, because there are no guarantees that the disease will not evolve resistance against them. Then, how can the transition from low-risk to high-risk disease be thwarted? And how can CRPC be treated without the ensuing drug resistance?

### 1.2. Benign Prostatic Hyperplasia 

BPH is the most common pathologic and clinical abnormality associated with the prostate gland. Indeed, histologic BPH lesions are encountered in men as young as in their 20s and 30s and the prevalence of the disease continues to rise as they age [26]. Although accurate estimates like those available for PCa are not available for BPH, epidemiological data indicate that asymptomatic BPH affects 50% of all men by the time they turn 50, and escalates to >90% in men 85 years or older [27,28]. While BPH is often histologic, a significant fraction of men have symptomatic forms of the disease which can result in lower urinary tract symptoms and even bladder outlet obstruction. It thus follows that BPH and PCa can coexist in the same patient. 

### 1.3. Coincidence of BPH and Prostate Cancer

Although PCa typically presents in men in their late 60s [29], the disease has been detected in men who are only in their 20s [30]. In fact, the coexistence of BPH and PCa has been known for >60 years. In an autopsy study of cadavers with and without PCa, Sommers [31] found that BPH was present in 80% of cadavers with PCa and only in 45% of cadavers without PCa. It therefore seems quite probable that most men diagnosed with PCa also have histologic BPH. Consistently, Ørsted et al. [32], who evaluated over 3 million Danish men, found that BPH was associated with an increased incidence of PCa as well as resulting mortality from the disease. However, the authors cautioned that their data underscoring the coincidence of the two diseases should not be used to infer causality between the two. On the other hand, Kulkarni and Getzenberg [33] have argued that there could be a causal relationship between the two diseases.

Regardless, however, it is quite imminent that most men diagnosed with BPH, and in particular symptomatic BPH, will have PCa within their prostate glands. In addition to the practically ubiquitous histologic association, both diseases share many commonalities in their etiologies including androgen dependence, smoking, a diet high in fat [34], and inflammation [35,36,37,38]. Finally, epidemiological studies are weighted towards the presence of BPH increasing the risk for a man to develop PCa in his lifetime [39,40]. Considered together, it seems reasonable to hypothesize that, at a mechanistic level, one or more common factor(s) may underlie the etiology of symptomatic BPH and PCa and if so, targeting such factor(s) would satisfy the unmet medical needs of these diseases that account for a significant portion of the healthcare burden worldwide, particularly in the industrialized world.

### 1.4. PAGE4, a Factor Common to Both BPH and PCa

In this section, we discuss how Prostate-Associated Gene 4 (PAGE4) may not only be the missing link between symptomatic BPH and PCa, but also a highly validated target for these burgeoning urologic diseases. 

**Figure 1:**
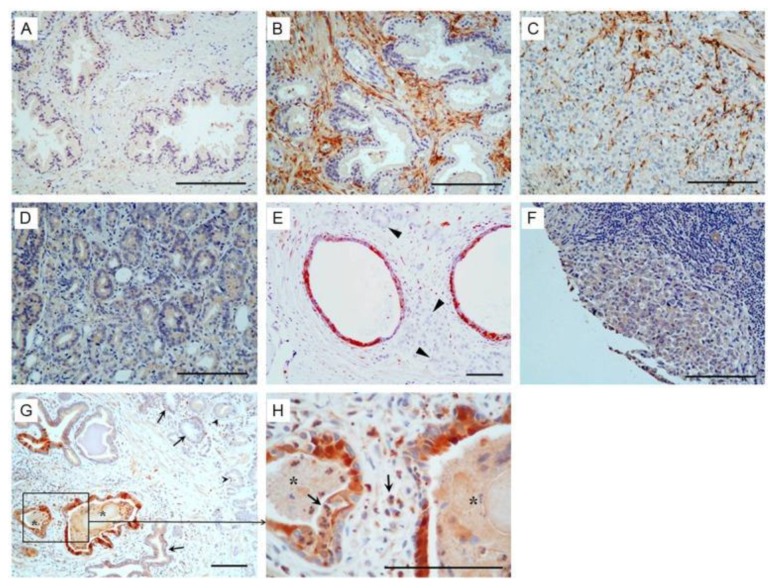
Immunohistochemistry analysis of Prostate-associated Gene 4 (PAGE4) in prostate cancer. (**A**) Negative staining in the normal prostate. (**B**) Intense staining shown in the stromal tissue in benign prostatic hyperplasia (BPH). (**C**) Positive staining in the stromal cells but negative in the cancer cells in some prostate cancer (PCa) specimens. (**D**) Moderate staining in the cancer cells but negative in the stromal cells in some PCa specimens. (**E**) Positive staining in the atrophic glands but negative in the cancer cells (arrowhead). (**F**) Negative staining in metastatic PCa. (**G**) Intense staining shown in cancer adjacent “normal” glands (asterisk) associated with inflammation but only moderate staining in the cancer cells (arrowhead). (**H**) High power view of boxed area in (**G**). Asterisk, proliferative inflammatory atrophy (PIA) lesions; arrows, inflammatory cells. Scale bars in all panels, 100 μm.

First, PAGE4 is a Cancer/Testis Antigen (CTA). The CTAs are a heterogeneous group of proteins that are by definition typically restricted to the testis, an immune-privileged organ, but are aberrantly expressed in cancer [41,42,43]. Furthermore, while PAGE4 is remarkably prostate-specific in men, its expression is highly dynamic. PAGE4 protein is highly upregulated both spatially and temporally in the prostatic epithelium and stromal cells of the fetal prostate up to 21 weeks, but is undetectable by 36 weeks [44]. In contrast, PAGE4 is undetectable in the normal adult prostate [45]. However, PAGE4 protein is highly expressed in the epithelial cells in Proliferative Inflammatory Atrophy (PIA) lesions and in high-grade Prostatic Intraepithelial Neoplasia (PIN) lesions [45], both of which are thought to be PCa precursors [46]. Furthermore, while PAGE4 protein expression is upregulated in primary (organ-confined, androgen-dependent) PCa, metastatic PCa specimens show no detectable PAGE4 protein [45,47,48] (Figure 1), which is in excellent agreement with the mRNA expression pattern (Table 1) [47]. These data suggest that PAGE4 may actually function as a proto-oncoprotein that is important in early development but is pathological if aberrantly expressed in the adult prostate gland. Consistent with this hypothesis, PAGE4 mRNA is significantly upregulated when somatic cells are reprogrammed to form induced pluripotent stem (iPS) cells but is undetectable in the parent somatic cells used in reprogramming [49]. Moreover, while knocking down PAGE4 expression results in cell death in vitro, its overexpression results in a growth advantage of PCa cells [50].

Second, PAGE4 protein is highly overexpressed in symptomatic BPH, which shares similarities with PCa at the molecular level, but not in asymptomatic BPH (also referred to as histologic or incidental BPH) [51]. In addition, unlike in the case of PCa where PAGE4 expression is observed in epithelial cells, in BPH, PAGE4 protein is predominantly expressed in the stromal and smooth muscle cells [51]. Considered together, the extensive spatiotemporal and cell-type specific expression data in the fetal and diseased gland suggest that PAGE4 appears to play important roles in both benign and malignant disease of the prostate, perhaps by modulating the effects of inflammatory stress.

Third, consistent with its role as a stress-response protein, PAGE4 is upregulated in response to a variety of stress inducers including inflammatory stress. Thus, exposing PCa cells to any environmental (drug treatment) or nutrient stress (glucose deprivation), or treating them with the proinflammatory cytokine tumor necrosis factor alpha (TNF-α), results in upregulation of PAGE4 both at the mRNA and protein levels [45]. Perhaps the most tantalizing evidence supporting PAGE4 as a molecular link between stress and PCa onset comes from the observation that PAGE4 is upregulated in normal prostate epithelial cells when exposed to the above-mentioned stress inducers [45]. The fact that PAGE4 is upregulated in PCa precursor lesions affected by inflammatory stress lends further credence to this argument. Indeed, inflammation appears to play an important role in both BPH, especially symptomatic BPH, and PCa [51,52,53]. In fact, as noted earlier, current epidemiological data indicate that >25% of all cancers are related to chronic infections and other types of unresolved inflammation and chronic inflammation is now regarded as an “enabling characteristic” of all human cancers including PCa [36,37,38]. 

Fourth, PAGE4 is phosphorylated at S9 and T51 by Homeodomain-Interacting Protein Kinase 1 (HIPK1), a component of the cellular stress–response pathway, and this doubly phosphorylated PAGE4 (HIPK1-PAGE4) functions as a strong potentiator of the oncoprotein c-Jun [54]. c-Jun is also a component of the stress–response pathway, and forms a heterodimer with members of the Fos family to produce Activator Protein-1 (AP-1). This group of early response transcription factors represents a paradigm for signal-responsive factors with important roles in the control of cell growth, apoptosis, and stress response [55]. The AP-1 factors are also upregulated in PCa [56] as well as in symptomatic but not asymptomatic BPH [52], underscoring the mechanistic significance of the PAGE4/AP-1 interactions in these diseases. Phosphorylation at T51 is necessary for the activity of HIPK1-PAGE4, because a mutation in this residue to an alanine abolishes its activity by >90% [54]. In addition to HIPK1, PAGE4 is also phosphorylated by CDC-Like Kinase 2 (CLK2), a dual-specificity kinase that phosphorylates serine-rich (SR) proteins of the spliceosome complex. But in contrast to HIPK1, CLK2 hyperphosphorylates PAGE4 at multiple S/T residues (including S9 and T51). Hyperphosphorylation by CLK2 attenuates rather than potentiates c-Jun transactivation [57]. Thus, in response to differential phosphorylation by the two kinases, PAGE4 could be remodeled to populate distinct conformational ensembles that may play an important role in regulating the phenotype of PCa cells in terms of their dependence on androgen (see below). Interestingly, while PAGE4, HIPK1, and CLK2 are all up-regulated in PCa, they are differentially expressed in PCa cells. Thus, CLK2, like PAGE4, is expressed in androgen-dependent PCa while HIPK1 is expressed both in androgen-dependent and androgen-independent PCa cells [57].

Fifth, like the majority of the CTAs, especially the CT-X subgroup [58], PAGE4 is a highly intrinsically disordered protein (IDP) [50]. Thus, the protein is characterized by lack of a rigid 3D structure and exists as an ensemble of interconverting conformations instead (hence, the ‘dancing protein cloud’ metaphor, a term coined by Vladimir Uversky [59]), which, if overexpressed, can lead to a pathological state [60]. However, detailed biophysical investigations revealed that the polypeptide has some local and long-range conformational preferences that are perturbed by phosphorylation, resulting in population shifts and altered intramolecular conformational dynamics [61]. Thus, the population of transient turn-like structures increases upon phosphorylation in a ~20-residue acidic region centered on T51. This central region becomes more compact and more negatively charged upon phosphorylation with increasing intramolecular contacts to basic sequence motifs near the N and C termini. Furthermore, although flexibility is decreased in the central region of the phosphorylated ensemble, the polypeptide chain remains disordered and highly dynamic overall. PAGE4 uses a transient helical structure adjacent to the central acidic region to bind c-Jun in vitro [61].

## 2. Targeting PAGE4 in Symptomatic BPH and Early-Stage ‘Low Risk’ PCa

### 2.1. Small Molecule Inhibitors (Disrupt PAGE4/AP-1 Interaction)

From the foregoing, it follows that detailed structure-function studies on PAGE4 and its interaction with AP-1 may yield additional insights that can potentially be used to identify small molecule compounds which disrupt the PAGE4/AP-1 complex. In silico studies using molecular dynamic simulations and computer-assisted drug design could further buttress such attempts and help screen for small molecule inhibitors. Similar reports in the literature have successfully identified small molecules against other IDPs that play important roles in chronic diseases [62], lending further credence to this strategy. The discovery of inhibitors of the onco-protein c-Myc [63,64]; the cell cycle regulator p27 [65]; and against nuclear protein 1 (NUPRI) [66], a stress protein involved in pancreatic cancer, are some recent examples of such endeavors. Thus, the availability of small molecule compounds or drugs could help treat and manage not only symptomatic BPH but also low-risk PCa patients, perhaps as a pre-emptive preventative measure. One potential issue comes to mind with regard to inhibiting PAGE4: Could low risk PCa progress to more aggressive forms in the absence of PAGE4? However, the fact that knocking down PAGE4 leads to cell death due to apoptosis is reassuring [50].

### 2.2. Immunotherapy

**Figure 2:**
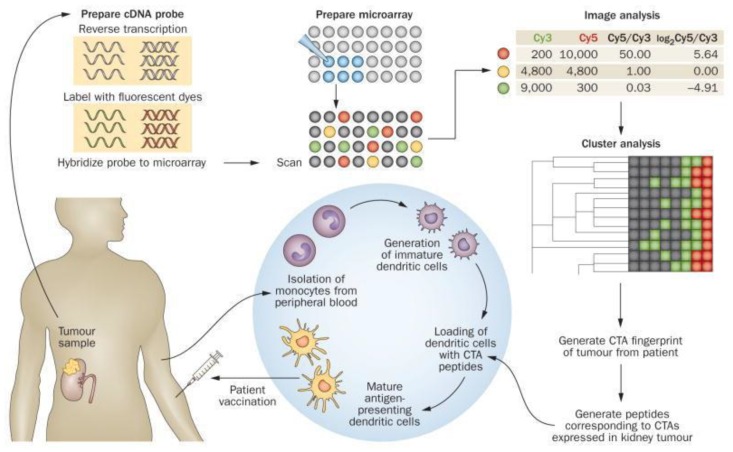
Schematic diagram of an immunotherapeutic approach to treating urological malignancies that utilizes immunogenic peptides corresponding to CTAs. A tumour sample is profiled for CTA expression using DNA microarrays. A CTA ‘fingerprint’ is generated and immunogenic peptides corresponding to the CTAs are generated that are used to pulse in vitro immature dendritic cells isolated from the patient. The pulsed cells mature into professional antigen-presenting cells, which are then injected back into the patient to trigger an immune response. Abbreviations: CTA, cancer/testis antigen; Cy3, cyanine 3; Cy5, cyanine 5. (From Kulkarni et al., 2012 [70]).

The efficacy of Sipuleucel-T (Provenge), an autologous cellular immunotherapy and the first immunotherapy product approved by the US FDA, has set a precedent for PCa immunotherapy [67,68,69]. This treatment approach is emerging as a promising method for chemotherapy-resistant PCa, and perhaps even early-stage disease. Indeed, accumulating evidence suggests that several CTAs including PAGE4 may also be good candidates for PCa immunotherapy [70]. In support of this possibility, Yokokawa et al. [71] reported the identification and characterization of a novel PAGE4 cytotoxic T lymphocyte (CTL) epitope, and the generation of an enhancer agonist of this epitope. T-cell lines generated from PCa patients using the agonist peptide showed high levels of lysis of PAGE4-expressing tumor cells and enhanced secretion of Interferon gamma (IFN-g), granzyme B, Tumor Necrosis factor alpha (TNF-a), Interleukin 2 (IL-2) and lymphotactin. Thus, these studies provide the rationale for the potential utility of the novel PAGE4 agonist epitope. As illustrated in Figure 2, one possible immunotherapeutic approach to treating PCa is to utilize PAGE4 immunogenic peptides. Per this paradigm, immunogenic peptides corresponding to the PAGE4 molecule are generated; they are used to pulse in vitro immature dendritic cells isolated from the patient. The pulsed cells mature into professional antigen-presenting cells, which are then injected back into the patient to trigger an immune response.

As is well known, therapeutic vaccination for cancer treatment depends on T-cell response to tumor antigen [72]. Therefore, the identification and characterization of immunogenic PAGE4 peptides, together with the identification of respective Human Leukocyte Antigen (HLA) class I antigen restriction, and the fact that PAGE4 expression is predominantly restricted to the diseased prostate, makes this approach a highly attractive one for PCa immunotherapy. However, several issues will still need to be addressed. For example, low expression levels of PAGE4 coupled with the notorious tumor heterogeneity can make this treatment option challenging. Second, heterogeneity in PAGE4 expression could also impair immunogenicity and immune recognition of cancer cells by the immune system, resulting in decreased vaccination efficacy. Finally, it is conceivable that tumour cell heterogeneity could lead to the emergence of PAGE-negative neoplastic clones, capable of escaping treatment-induced and PAGE4-specific immune surveillance. 

## 3. Reinstituting Sustained PAGE4 Expression in Advanced PCa

As mentioned above, HIPK1-phosphorylated PAGE4 (HIPK1-PAGE4) binds to AP-1 and potentiates c-Jun, but CLK2-phosphorylated PAGE4 (CLK2-PAGE4) has reduced affinity to AP-1 and attenuates c-Jun. These opposing activities are governed by the conformational dynamics of the PAGE4 ensemble. CLK2-PAGE4 adopts a more expanded and open conformation as compared to HIPK1-PAGE4 and nonphosphorylated PAGE4 (WT-PAGE4), as determined by multiple experimental techniques [57]. 

**Figure 3:**
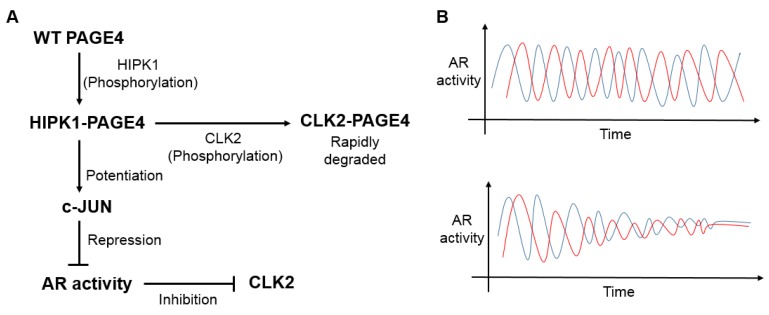
Cell-to-cell heterogeneity and dynamic variations in Androgen Receptor (AR) activity in an isogenic population. (**A**) Regulatory circuit formed by interactions among Prostate-associated Gene 4 (PAGE4), Homeodomain-Interacting Protein Kinase 1 (HIPK1), CDC-like Kinase 2 (CLK2), c-Jun, and AR activity—a negative feedback loop. (**B**) Top panel, in absence of any therapeutic intervention, this negative feedback loop can lead to intracellular oscillations in AR activity. Different colored curves show oscillatory dynamics for two cells in a given isogenic population; these oscillations need not be synchronized, thus, generating cell-to-cell heterogeneity. Bottom panel, upon application of Androgen Deprivation Therapy (ADT) (or periods of AR inhibition during Intermittent Androgen Deprivation Therapy (IAD)), these oscillations can get quenched.

Interestingly, CLK2 levels can be lowered by AR activity, which in turn is repressed by c-Jun transactivation. Therefore, enhanced HIPK1-PAGE4 can indirectly increase CLK2 levels and promote its hyperphosphorylation to CLK2-PAGE4. These interconnections can, thus, form a negative feedback loop, which may give rise to oscillations in intracellular levels of the different conformational ensembles of PAGE4 as well as AR activity, as predicted by a mathematical model [57] (Figure 3). It is important to note that the population of a given PAGE4 conformational ensemble is dependent on its phosphorylation status. In other words, cell-to-cell variability due to differential phosphorylation of PAGE4 can promote phenotypic heterogeneity. Consequently, androgen-dependence of a given cell can be a time-varying function, i.e., a cell can exhibit various extents of androgen-dependence at different time points. Moreover, cells in a population need not oscillate synchronously, generating non-genetic heterogeneity in an otherwise clonal population—an insuperable clinical challenge [73]. These oscillations can be dampened by ADT or even Intermittent ADT (IAD) [74]. However, the oscillations can be reinstituted if ADT is abrogated (or if IAD is used instead) suggesting that PCa cells can potentially transition from an androgen-resistant to and androgen-sensitive phenotype reversibly. It should be noted that these oscillations are pertinent only for cells that do not undergo cell death in response to ADT/IAD. This argument is further strengthened by a recent report [75] demonstrating that differential phosphorylation of the heat shock factor 1 (Hsf1) generates cell-to-cell variation, and that this variation rather than the absolute Hsf1 activity promotes phenotypic plasticity in yeast.

Results from a recent preclinical study employing patient-derived xenografts (PDX) in mice [76] reinforce the above-mentioned observations of reversible cellular plasticity. This study showed that when tumor specimens taken from clinically castration-resistant patients were implanted into intact mice, these resected tumors resulted in androgen-sensitive PDXs that could be maintained by serial passage in intact male mice. Upon castration, these tumors initially responded but subsequently grew as castration resistant (CR) lines that could be serially maintained in castrated male mice indicating that cells could transition between androgen-sensitive and androgen-resistant phenotypes reversibly, and that these transitions may be influenced by microenvironmental conditions such as the availability of androgen. Furthermore, tumors may contain different clones with varying androgen sensitivity and thus, varying fitness to different environmental conditions, suggesting that besides reversible phenotypic switching, additional mechanisms may drive androgen resistance such as, the outgrowth or dominance of one ‘clonal’ phenotype over the other [77]. While the oscillations in the PAGE4/AP1-1/AR circuit and their quenching in response to therapy remains to be experimentally validated, the potential implications of conformational dynamics of PAGE4 in giving rise to subpopulations with varying sensitivities to these therapies are quite imminent. Of note, a recent report by Gallaher et al. [78] who investigated drug resistance in PCa, provides credence to this argument. By using drug dose modulation or treatment vacations, the authors observed that adaptive therapy strategies can control the emergence of drug resistance by spatially suppressing less-fit-resistant populations in favor of treatment-sensitive ones.

## 4. Conclusions and Outlook

**Figure 4:**
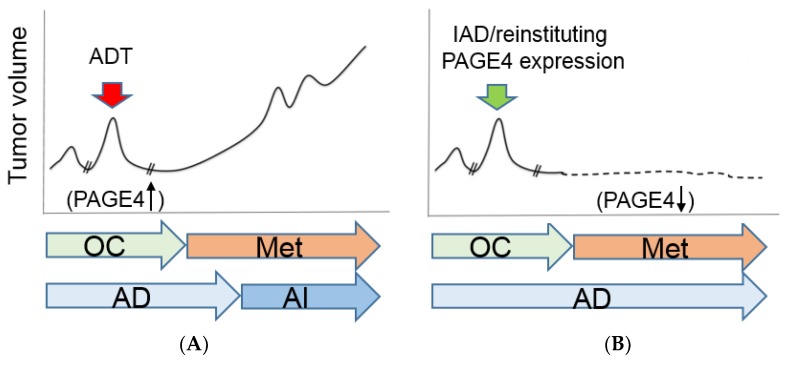
Schematic diagram indicating a new type of precision medicine for prostate diseases. (**A**) In symptomatic BPH and in organ-confined prostate cancer where PAGE4 is upregulated, inhibiting PAGE4 using small molecule inhibitors may be a novel therapeutic strategy to treat these diseases. (**B**) Reinstituting PAGE4’s sustained expression either by intermittent ADT (IAD) or protein expression in metastatic prostate cancer, where it is downregulated, is likely to subvert or attenuate the emergence of CRPC. OC, organ-confined; Met, metastatic; AD, androgen-dependent; AI, androgen-independent.

In this perspective, we have shed new light on a prostate-specific protein that has remained relatively under-appreciated despite the enormous promise it holds as a biomarker, as a therapeutic target, and as a potential therapeutic itself. As far as we are aware, there is no other molecule that fits these criteria in any cancer, much less in PCa. We have summarized results indicating that inhibiting PAGE4 using small molecule inhibitors may be a novel therapeutic strategy for symptomatic BPH, and for low-risk, androgen-sensitive PCa, where it is highly upregulated. We also indicated that reinstituting PAGE4’s sustained expression in metastatic PCa, where it is downregulated, is likely to subvert or attenuate the emergence of CRPC. Toward this end, multiple avenues such as oncolytic viruses [79,80] or nanoparticles [81,82] to deliver protein therapeutic payloads may be contemplated. Alternatively, IAD may present an unprecedented opportunity to tackle high-risk PCa (Figure 4). While the jury is still out, and a clear advantage of IAD over ADT remains equivocal, proponents argue that IAD can abrogate some of the undesirable side effects and costs associated with continuous ADT while improving the quality of life for PCa patients [3].

**Figure 5:**
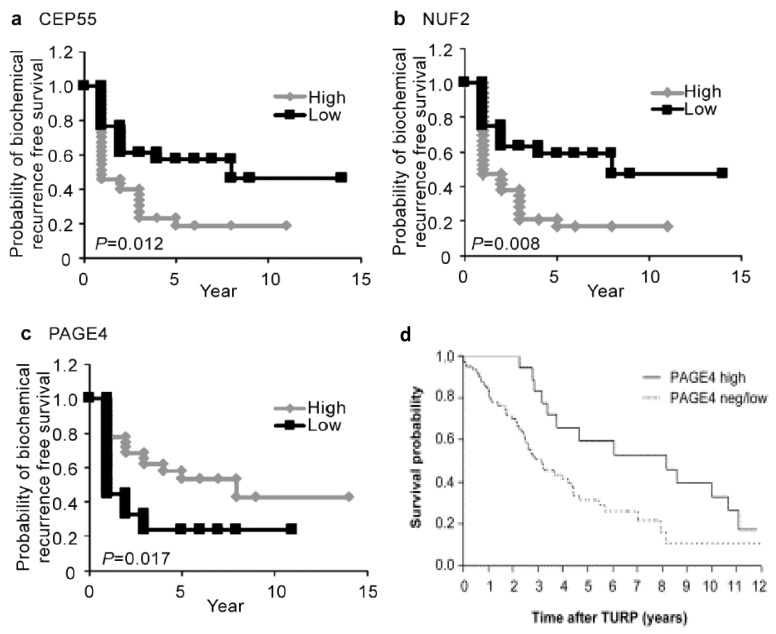
Kaplan–Meier analyses. Kaplan–Meier curves showing biochemical recurrence-free survival against time after radical prostatectomy stratified by the mRNA expression of (**a**) CEP55, (**b**) NUF2 and (**c**) PAGE4 (high versus low groups dichotomized by median value). (From Shiraishi et al., 2012). (**d**) PAGE4 levels correlate with survival of patients with hormone-naive PCa. Overall survival of patients with hormone-naive PCa after TURP for local advanced obstructive PCa stratified for high versus negative/low (neg/low) epithelial PAGE4 levels on the advanced PCa TMA (third quartile of mean epithelial PAGE4 intensity was set as the cut-off level) (From Sampson et al., 2012).

In further support of reinstituting PAGE4 expression in advanced disease, two lines of tantalizing evidence are noteworthy. First, in a preclinical study, compared to vector only control tumors, the growth of PCa xenograft tumors overexpressing PAGE4 was attenuated when the host was castrated [83]. These data tend to suggest that the presence of PAGE4 attenuated PCa growth in an androgen-depleted background that may, at least conceptually, mimic CRPC. Second, clinical studies have revealed that higher levels of PAGE4 are an indicator of better PCa prognosis. Thus, in hormone-naive PCa, the median survival of patients with high PAGE4 expression was 8.2 years compared with 3.1 years for patients with PAGE4 negative or with low PAGE4 expression [83]. Conversely, loss of PAGE4 correlated with poor overall survival in hormone-naive PCa patients. Consistently, a lower level of PAGE4 mRNA correlated with reduced incidence of biochemically recurrent PCa, although it was not an independent predictor of biochemical recurrence [48] (Figure 5). 

Furthermore, a serum-based enzyme-linked immunosorbent assay using a monoclonal antibody that can detect PAGE4 at a low ng/mL level can distinguish patients with symptomatic and asymptomatic BPH with high sensitivity and specificity [84]. Although the presence of PCa in these men did not appear to alter PAGE4 levels, this study together with some others [85], highlights the potential of biomarker-based screening and gene-specific therapies as tools to risk stratify patients with BPH and identify those with symptomatic or medically resistant forms. In summary, though additional studies will be needed to fully adjudicate the full potential of PAGE4, it is tempting to conjecture that, in the future, PAGE4 could serve as a theranostic marker so that appropriate treatment modality (inhibition versus sustained expression) for each patient could be tailored based on serum PAGE4 levels heralding a new type of precision medicine for prostate diseases. Thus, PAGE4, a hitherto unappreciated molecule, may be the dark horse in the race for an effective therapy for these increasingly prevalent prostatic maladies.

## Figures and Tables

**Table 1:** PAGE4 expression is down regulated in metastatic prostate cancer; CTA, Cancer/Testis Antigen (CTA).

CTA	BPH	Primary PCa	Met PCa
PAGE4 (205564_at)	6732.5	5702.8	56.6
CSAG2 (220445_s_at)	16.6	20.6	1026.4
MAGEA2 (214603_at)	46.7	42.7	1343.9
MAGEA6 (214612_x_at)	30.5	16.2	2785.1
MAGEA12 (210467_x_at)	34.9	52.9	1168.4
TBP (203135_at)	608.66	577.58	882.2
